# The Lessons of a Rebuilding Scheme

**Published:** 1912-11-23

**Authors:** 


### THE LESSONS OF A REBUILDING SCHEME.

### Mr. H. Jennings on the Chelsea Hospital Extension.

It is hardly sufficiently realised that if the story of every rebuilding scheme were published authoritatively much useful experience could be collected for the assistance of hospital managers, who always have to be considering projects of a similar kind, and often fall into errors from a want of interchange of experiment and knowledge. With a view, therefore, to securing a record of the system on which the new Chelsea Hospital for Women is being built our Commissioner visited Mr. Herbert Jennings to find out from him the methods adopted and the particular problems which had to be faced.

"Let me preface my remarks," Mr. Jennings began, "by reminding you that it was our miserable accommodation for out-patients and our inadequate number of beds which forced on my Committee a consideration of the possibility of rebuilding. So much operative work is done in the Chelsea Hospital, and the want of proper sterilising accommodation is so great, that what we have really been trying to do, to put it briefly, has been to compress a quart into a pint pot. Some years ago it was known that site changes were pending in Chelsea, but when we approached Lord Cadogan in the matter we had no reason to hope for the striking generosity which has now placed at our disposal a free site in Arthur Street, between the two great thoroughfares of the King's and Fulham Roads. The long narrow frontage of the site is more than compensated by its freedom from noise, which is what our present position has never possessed. The site is long and narrow, and will lend itself excellently to the buildings to be erected on it. As to the question of cost. The financing of the scheme owes a great deal to the generosity of the Trustees of the Zunz Bequest, who gave £2,000 a year or two ago, and have now followed that up with a promise of £10,000 towards the rebuilding scheme. Then, again, thanks to the generosity of Mr. Dyer Edwardes, who has foregone a mortgage of £4,000, the existing site is free to be sold for the advantage of the hospital. With these satisfactory preliminaries, we went to King Edward's Hospital Fund, and proceeded to obtain sketch plans from Mr. Keith Young, F.R.I.B.A. The sittings of the Special Committee then began."

### The Plan of Nursing Units.

"What were the chief requirements?"

"First of all my Committee desired to raise the number of beds from fifty to seventy-six, including four beds for isolation cases. It was found convenient to divide the seventy-two ordinary beds into four blocks of eighteen each, and to call each of these a nursing unit. A nursing unit would be under one sister, and would comprise three wards of four beds each, and one of six. Each unit would have ward kitchen, lavatories, store cupboards, and other necessary adjuncts. One of the units will be named the "Annie Zunz," after the lady in whose honour the Trust was formed. The narrowness of the site, which runs north and south, made it impossible to design the wards at right angles to the frontage, so the nursing units are placed longitudinally on either side of the central block."

"Why was this arrangement of nursing units decided on, and what are the advantages which can be claimed for it?"

"At first it was contemplated to divide seventy-five beds into five separate units, according to the number of the in-patient staff. Then it was found more economical to build four nursing units, making each unit a little larger, and this plan also promised more economical administration. Briefly, the basement contains the store, porter's rooms, and kitchen on the south, together with the out-patient department at the north end. On the ground floor are the board room, secretarial offices, and resident medical officers' rooms to the north, and the matron's quarters and the nurses' dining rooms to the south. The isolation accommodation is at the back. On the first floor is a nursing unit at either extremity, with the theatre at the back. The nurses' home is separate from the hospital, and divided from it by a garden. Thanks to our nurses' reputation for kindness to the patients, of which the ladies interested in the hospital are always receiving striking testimony, the Ladies' Committee are making a special effort on behalf of the home."

### Wall Surfaces, Floors, and Materials.

"Can you give me any details of the Committee's investigations and decisions in matters of detail?"

"I am coming to that now. So many points in hospital construction are matters of debate that it was deemed essential to collect the experience of other institutions before contracts were placed and materials decided on. With this end in view the members of the Committee paid a series of visits to the London, St. Thomas's, Metropolitan, Hampstead, Putney, and Copse Hill Hospitals, some of these being institutions only recently built or rebuilt. They paid special attention to wall surfaces and floors. As regards the former, experience seemed to teach that a dado of opalite tiles, with good painted plaster above, was the least absorbent and best wearing material for the walls compatible with due economy. As to the ward floors, after much attention had been paid to patent floors of various kinds, opinion seemed to favour linoleum laid on concrete for the floors of the ward, terrazzo laid in panels for the corridors, while terrano recommended itself for the out-patient department."

"Then as regards the tenders for building?"

"I understand that, although it is the practice to invite tenders for the general building work, yet sometimes the subsection of special work, like electric lighting, sanitary fittings, etc., are put in the hands of one particular firm without inviting tenders. There may be some advantage in this system, but my Committee think they are far more than outweighed by disadvantages which are obvious from the economic standpoint. They felt strongly that there is no monopoly of good work, and also that the known absence of rivals which a general contract secures tends to enhance the prices asked for by firms who have no check on their estimates in the form of rival competitors.

"I have ventured to enter into these details at some length as an indication of what may be found very useful by other hospital committees situated like ours. Our representatives were always welcomed on their visits, given every facility for seeing what they wished to investigate, and afforded much practical information on points that arose out of those immediately in question. My account is only a tentative movement in this direction, but, if it were followed up by hospitals generally, an immense amount of careful information could be compiled and made accessible."

